# Influenza A-Induced Myocarditis and Persistent Complete Heart Block in Pregnancy

**DOI:** 10.7759/cureus.90641

**Published:** 2025-08-21

**Authors:** Zeina Othman, Hannah Catton, Tandip Mann, Betsy Dwyer, Constantinos G Missouris, Amit K J Mandal

**Affiliations:** 1 Department of Obstetrics and Gynaecology, Wexham Park Hospital, Frimley Health National Health Service (NHS) Foundation Trust, Slough, GBR; 2 Department of Anaesthesiology, Wexham Park Hospital, Frimley Health National Health Service (NHS) Foundation Trust, Slough, GBR; 3 Department of Medicine, Wexham Park Hospital, Frimley Health National Health Service (NHS) Foundation Trust, Slough, GBR; 4 Department of Clinical Cardiology, University of Nicosia Medical School, Nicosia, CYP

**Keywords:** complete heart block, influenza a, myocarditis, pacemaker, pregnancy

## Abstract

Cardiovascular implications of respiratory viruses are often overlooked. Arrhythmia is a rare manifestation of influenza A and is usually self-terminating after the acute phase. Only a handful of cases of complete heart block (CHB) associated with influenza A have been reported. We present the case of a healthy 33-year-old primigravida who developed CHB at 20 weeks’ gestation after contracting influenza A, requiring hospitalisation. Thereafter, the patient remained asymptomatic throughout the prepartum period despite persisting CHB and bradycardic escape rhythm. Delivery was successfully managed with spinal anaesthesia and close haemodynamic monitoring. Postpartum cardiac imaging confirmed myocarditis. CHB with a narrow escape rhythm persisted after delivery. The emergence of symptoms and a blunted heart rate response to exercise necessitated elective permanent pacemaker (PPM) implantation after careful consideration. This case underscores the importance of cardiac evaluation in pregnancy, particularly after seasonal influenza, and highlights the challenges in diagnosing and managing CHB within this context.

## Introduction

Influenza carries an underappreciated burden of cardiovascular complications, including arrhythmia and heart failure through myocarditis. Ten percent of viral myocarditis is attributable to influenza. Though typically self-limiting, influenza may rarely precipitate significant conduction abnormalities [1]. While sinus bradycardia and low-grade atrioventricular (AV) blocks are more frequently encountered, complete heart block (CHB) remains exceedingly rare.

CHB in pregnancy is an uncommon but clinically important entity that requires a multidisciplinary approach. Most documented cases are congenital, and 30% remain undiagnosed until adulthood [2-4]. Acquired CHB is exceptional and usually associated with older maternal age or secondary causes such as infection, autoimmune, or infiltrative disease [4].

Given its scarcity, guidance is based on consensus opinion and anecdotal experience [4,5]. This report adds to the limited literature surrounding CHB in pregnancy precipitated by influenza A and explores diagnostic ambiguity, anaesthetic considerations, postpartum management, and timing of permanent pacemaker (PPM) implantation.

## Case presentation

A 33-year-old primigravida at 20 weeks’ gestation was hospitalised with confirmed influenza A. A ventilation-perfusion scan revealed a small-volume peripheral pulmonary arterial thrombus, for which she was prescribed low-molecular-weight heparin until six weeks postpartum. There was no relevant medical or family history. Electrocardiogram (ECG) demonstrated CHB with narrow escape rhythm at a rate below 60 beats per minute (bpm). Laboratory investigations were notable for N-terminal-pro-B-type natriuretic peptide of 2,133 ng/L (0-299 ng/L), and troponin I peaked at 87 ng/L (0-15 ng/L). Thyroid function was normal. Transthoracic echocardiography (TTE) confirmed normal cardiac structure and function. Undiagnosed congenital CHB was the presumed diagnosis, and pacemaker decisions were deferred until after delivery, since asymptomatic.

**Figure 1:**
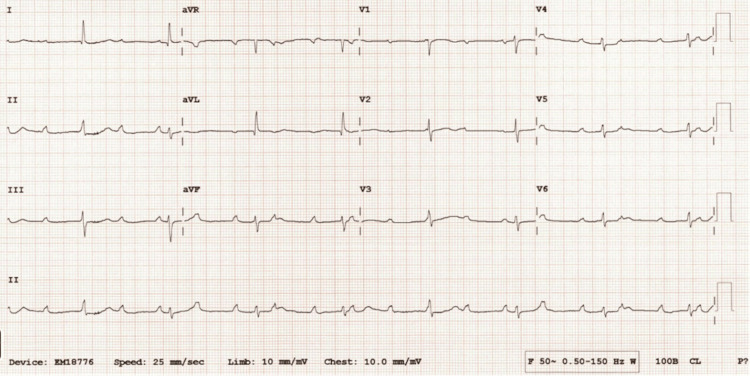
Resting 12-lead ECG demonstrating CHB with narrow escape at 40 bpm. ECG: electrocardiogram; CHB: complete heart block

At 39 weeks’ gestation, she was admitted in advance of an elective caesarean section for breech presentation. ECG demonstrated CHB with a narrow QRS escape rhythm at a rate of 40 bpm (Figure 1). Repeat TTE was grossly normal.

She reported pre-influenza heart rates of around 80 bpm consistently captured on her smartwatch. There was no untoward symptomology. Clinical examination was normal with a gravid uterus. A multidisciplinary team comprising anaesthetists, obstetricians, and specialist physicians was gathered to provide peripartum care. Narrow escape rhythm and absence of symptoms were reassuring.

On the day of delivery, transcutaneous pacing was on standby, and an arterial line was inserted. She underwent spinal anaesthesia with a block at L3-4. Phenylephrine infusion (100 mcg/mL) was administered at a deliberately low rate of 2 mL/hr to minimise vagotonia. The usual infusion rate would normally be 30 mL/hr prepartum and reduced to 10 mL/hr postpartum in an effort to support maternal blood pressure and foetal perfusion. Intraoperatively, after delivery of the baby, she developed profound bradycardia, which was readily responsive to a single 600 mcg bolus of atropine. The procedure was otherwise uneventful, and external pacing was not required. Cardiotocography and neonatal heart rate were within the normal range, and the baby was healthy. Comprehensive maternal haematologic and biochemical panels including autoimmune and extended viral screens, serum angiotensin-converting enzyme levels, and QuantiFERON Gold were unremarkable.

At two months postpartum, cardiac magnetic resonance imaging (CMR) demonstrated mild biventricular dilatation with preserved systolic function and subepicardial inflammation and fibrosis in the basal inferior segment of the left ventricle. The pattern of late gadolinium enhancement and prominence of myocardial oedema and inflammation were consistent with non-ischaemic dilated cardiomyopathy and myocarditis rather than peripartum cardiomyopathy. These findings supported a unifying diagnosis of CHB related to influenza A-induced myocarditis.

**Figure 2:**
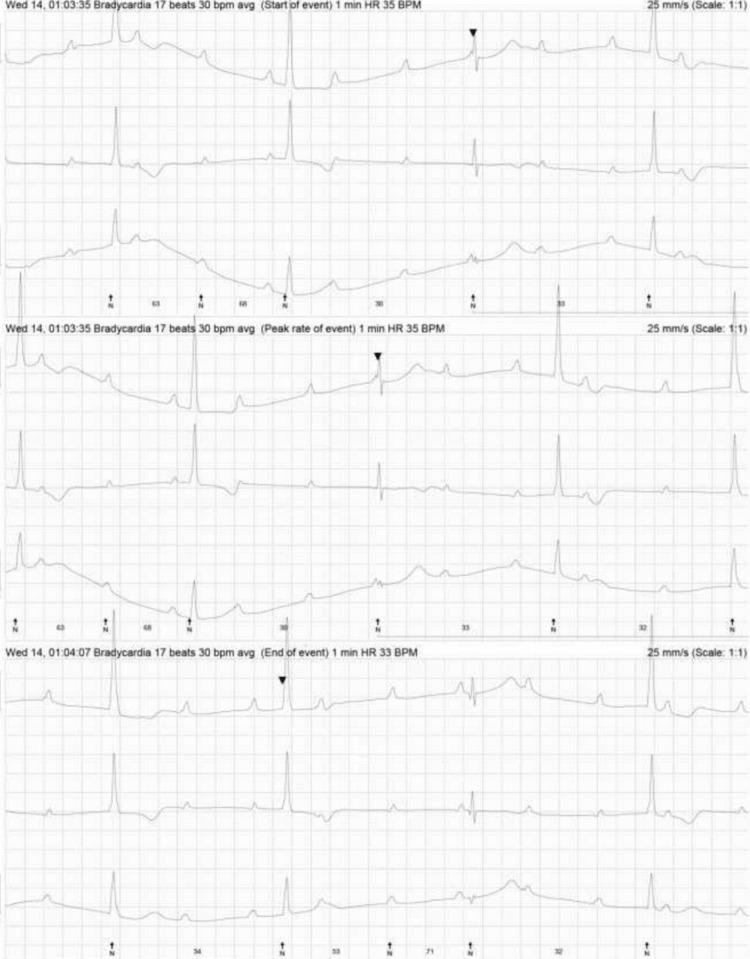
Holter excerpts demonstrating CHB with very bradycardic escape (<30 bpm). CHB: complete heart block

At three months postpartum, Holter monitoring demonstrated CHB with a narrow escape rhythm (Figure 2) and a mean heart rate of 36 bpm (range: 28-74 bpm). A stress test was abandoned after six minutes of the Bruce protocol due to dyspnoea. There were blunted heart rate and blood pressure responses to exercise. A maximal heart rate of 72 bpm represented 38% of the maximal age-predicted heart rate.

The patient was thereafter referred to a tertiary electrophysiology centre. She remained in CHB and reported unaccustomed dyspnoea. Expert opinion was that CHB was related to influenza A-induced myocarditis, albeit without huge scarring, and not vagally mediated. Peripartum cardiomyopathy was thought unlikely given the timeline and CMR tissue characterisation. Considerations were made for future pregnancies and general anaesthesia. Despite numerous box changes that would be required over her lifetime, consensus was to offer dual-chamber PPM implantation with left bundle branch area pacing (LBBAP).

**Figure 3:**
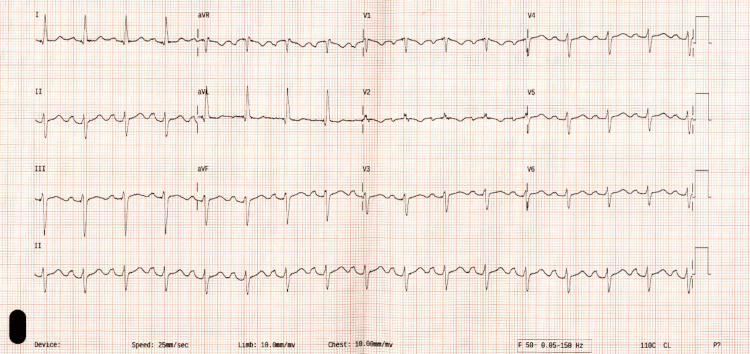
Resting 12-lead ECG demonstrating atrial sensed ventricular paced rhythm. ECG: electrocardiogram

At six months postpartum, the procedure was performed with CHB and narrow escape continuing as an intrinsic rhythm. Follow-up device interrogation after six weeks established pacemaker dependence. On review at one year postpartum, she was asymptomatic with no signs of heart failure, and cardiac biomarkers were normal. ECG demonstrated an atrial sensed ventricular paced rhythm (Figure 3).

## Discussion

CHB in pregnancy is rare, with an incidence of one in 20,000 live births [2]. It is usually congenital, presenting in childhood. When subclinical and diagnosed during pregnancy, management becomes complex [3,5], particularly with the materialisation of symptoms.

During pregnancy, the cardiovascular system adapts to support increased maternal and foetal demands. Physiological modifications involve increased blood volume, cardiac output, and heart rate, alongside a reduction in blood pressure and systemic vascular resistance. These expected cardiovascular changes can unmask latent conduction system disease [3,6], and chronotropic incompetence can bring about decompensation during labour [6]. Stable nodal or high junctional escape rhythms are usually well tolerated, but there remains an inherent risk of heart failure, impactful bradyarrhythmia, and asystolic arrest.

The pathophysiology of CHB involves disruption at various levels of the conduction system. An AV nodal block typically results in narrow QRS complexes and higher escape rates, whereas an infranodal block leads to broad QRS complexes and more significant haemodynamic compromise [2]. In our patient, dynamic stability, absence of symptoms, narrow escape rhythm, and response to atropine were consistent with AV nodal involvement.

Influenza A and B are common seasonal flu viruses. Influenza A is generally considered more severe with a heightened risk of viraemic complications. Influenza A is a rare but documented cause of acquired CHB through myocardial involvement. Penetration of cardiomyocytes and viral replication triggers myocardial inflammation, which may induce fibrosis and apoptosis, resulting in myocardial scarring and arrhythmogenic substrate [1]. Most influenza A-associated rhythm disturbances are transient, and only a handful of cases of persisting CHB requiring PPM implantation are reported [1]. To our knowledge, this is the first case of CHB and confirmed influenza A-induced myocarditis in the context of pregnancy requiring PPM implantation.

CHB in pregnancy requires coordinated management and preoperative planning with well-defined indications for external pacing, sedation, and conversion to general anaesthesia. Vaginal delivery is generally safe, and active second-stage shortening is recommended to prevent Valsalva-induced bradycardia [4]. Caesarean section is reserved for obstetric indications. Spinal anaesthesia at a level below T5 minimises sympathetic blockade and reduces risks of bradycardia and vasodilatory hypotension. Sympathomimetics such as phenylephrine, isoprenaline, or ephedrine are, therefore, frequently utilised, but reflex vagotonic effects must be monitored. Incremental dosing and invasive monitoring help maintain haemodynamic stability [2]. In our patient, phenylephrine was chosen because of our familiarity with the agent and managing any associated bradycardia. In addition, several randomised controlled trials and meta-analyses suggest that prophylactic phenylephrine infusion carries better foetal acid-base outcomes when compared to other sympathomimetics [7]. Further, the baroreceptor-mediated reflex bradycardia is mild, predictable, and readily reversed with glycopyrrolate or ephedrine. Although glycopyrrolate may be less effective (but just does enough), as a quaternary amine, there is limited placental transfer. In our patient, atropine, a tertiary amine that crosses the placenta easily, was administered because efficacy was particularly important, and the baby was delivered at the time of treatment.

Contextually, our case was myocarditis rather than peripartum cardiomyopathy, which generally has a better prognosis. Markers of myocardial damage were elevated on initial presentation, and with hindsight, this was a clinical diagnosis of myocarditis rather than silent congenital CHB. Moreover, the patient was clear that her heart rate had been within normal limits prior to hospitalisation, but remained consistently around 40 bpm on photoplethysmography thereafter. As Sir William Osler taught, 'Listen to your patient, (s)he is telling you the diagnosis' [8].

In asymptomatic younger patients, expectant observation is justified. PPM implantation in our patient was carefully deliberated, considering her age and numerous box changes that would be required during her lifetime. The decision to pace was based on myocardial scarring on CMR, persistent CHB with blunted heart rate response to stress, and postpartum symptom burden. In order to mitigate the risk of adverse cardiac events during future pregnancy and illness, dual-chamber PPM implantation, specifically with LBBAP, was considered appropriate. LBBAP is an emerging mode of conduction system pacing that guarantees left ventricular and, therefore, electrical synchrony and avoids adverse haemodynamics of right ventricular pacing [9]. By mimicking the natural conduction system, LBBAP achieves more physiological pacing with a narrow QRS duration and improved stability with better long-term pacing thresholds, ideal for young patients as in this case.

## Conclusions

The case adds to the evidence linking influenza A viraemia to serious conduction disorders such as CHB. It highlights the importance of cardiac assessment in pregnant patients with influenza, particularly when symptoms are severe. In this instance, timely multidisciplinary collaboration enabled safe delivery and facilitated optimal onward care. Subsequent investigations and clinical course mandated PPM implantation. Emphasis is laid upon the importance of perioperative planning, postpartum evaluation, and personalised pacing decisions.
